# Digital Physical Activity Interventions for Mental Health Promotion of and Reduction in Addictive Behaviors: Integrative Comprehensive Review with a Focus on Personalization and Implementation

**DOI:** 10.3390/ijerph23060703

**Published:** 2026-05-26

**Authors:** Pedro Morouço, Eduardo Ramadas

**Affiliations:** 1 Department of Research, VillaRamadas International Treatment Centre, 2400-121 Leiria, Portugal; jeduardo@villaramadas.com; 2 National Program for Physical Activity Promotion, Directorate-General of Health, 1049-005 Lisbon, Portugal

**Keywords:** behavioral change, relapse prevention, craving, self-monitoring, implementation and equity

## Abstract

**Highlights:**

**Public health relevance—How does this work relate to a public health issue?**

- Mental disorders and addictive behaviors are major contributors to the global burden of disease, and physical inactivity is a modifiable risk factor with strong public health relevance.
- Digital delivery can expand the reach and continuity of physical activity promotion for vulnerable populations, but evidence is fragmented across mental health and addictions.

**Public health significance—Why is this work of significance to public health?**

- This review synthesizes digital physical activity interventions by technology type and active ingredients, clarifying what is currently supported and where evidence is still limited.
- It identifies critical gaps (short follow-up, heterogeneous engagement metrics, and limited evidence in addictions) that constrain scalability and real-world effectiveness.

**Public health implications—What are the key implications or messages for practitioners, policy makers and/or researchers in public health?**

- Programs should prioritize feasible prescriptions, self-monitoring with actionable feedback, and low-friction implementation, with explicit attention to equity, privacy, and safety.
- Research should move toward longer follow-up, harmonized outcomes (mental health and addiction-related when applicable), and staged personalization grounded in transparent tailoring logic.

**Abstract:**

Digital interventions can increase the reach and continuity of physical activity promotion, but evidence remains fragmented across mental health and addictive behaviors. We conducted a comprehensive integrative review supported by structured searches (2015–2026) in biomedical, psychological, multidisciplinary and technology-oriented databases, complemented by backward/forward snowballing. Eligible studies included digital interventions in which physical activity (or sedentary reduction) was a core component and those that reported mental health outcomes (e.g., depression, anxiety, stress, and well-being) and/or addiction-related outcomes (e.g., craving, consumption, lapses/relapse, and treatment retention). We synthesized findings thematically by intervention typology (apps, wearables, hybrid models with human support, and adaptive approaches) and by key active ingredients (goal setting, self-monitoring, feedback, reinforcement, planning, and engagement strategies). Overall, most studies targeted mental health outcomes and used app-based multicomponent programs, sometimes complemented by wearables, with generally short follow-up and heterogeneous engagement metrics. Evidence in addictions was more context-specific and concentrated in alcohol treatment and opioid agonist therapy settings, supporting feasibility and a plausible role for physical activity as a coping strategy. Advanced personalization frameworks (EMA/EMI/JITAI) provide a clear implementation pathway but are less consistently operationalized when physical activity is the central therapeutic component. This review highlights practical design recommendations and research priorities for scalable, safe, and equity-oriented digital physical activity interventions in mental health promotion and relapse prevention.

## 1. Introduction

Physical activity is a central determinant of health and well-being. The evidence is consistent in the prevention of and reduction in depressive and anxious symptoms, at different ages and in different contexts [1,2]. It also improves sleep, stress regulation and quality of life. Therefore, it is a relevant instrument for the promotion of mental health within the public health domain [3].

Mental disorders and addictive behaviors continue to contribute markedly to the global burden of disease [4,5]. Comorbidity is frequent and clinically relevant. Depression and anxiety are associated with a higher risk of consumption and a higher probability of relapse [6]. Chronic stress and sleep disruption often appear as common pathways. Craving, in turn, is a core mechanism. It is not static. It fluctuates throughout the day and is sensitive to context, emotions and environmental stimuli [7].

Physical activity can act on several processes that are relevant in this domain. It can reduce stress reactivity and improve sleep architecture [8]. It can increase self-efficacy and self-regulation capacity [2]. In some populations, it can also modulate reward circuits and contribute to greater inhibitory control [9]. In addition, when it is structured and sustained over time, it can create alternative routines and reinforce social support, with an impact on maintaining change [1,3].

Despite these advantages, there is a recurring problem. Adherence is limited, and maintenance is difficult, especially in people with psychological distress, greater impulsivity or less social support [10,11]. This challenge is particularly evident in contexts of vulnerability, where logistical and economic barriers are strongest [10]. Thus, the question is not just whether physical activity “works”. It is also how to ensure that it is initiated, maintained and integrated into real care and health promotion pathways.

Meanwhile, digital technologies offer a practical opportunity to respond to part of this challenge [12]. They can increase reach and continuity through apps, platforms, and hybrid models with remote monitoring [13]. They facilitate self-monitoring, goal setting, and feedback. They can also incorporate coaching and social support, reducing barriers to access. An additional, increasingly relevant value is the possibility of real-time data collection (brief self-report combined with passive sensing), which can improve characterization of within-day dynamics and inform the design of more context-aware interventions, rather than constituting a direct benefit per se [14,15].

In this context, the paradigm of real-time adaptive interventions, often referred to as Just-In-Time Adaptive Interventions (JITAIs), is of particular interest [16]. The principle is to deliver the right support, at the right time and in the right context. This logic is especially suited to dynamic risk such as stress, sleep deprivation, and increased craving [17]. An intervention can identify windows of greater vulnerability and suggest physical activity micro-interventions, adjusting goals and strategies responsively [18]. It can also reinforce protective behaviors when the risk is lower, supporting maintenance in the medium term [16].

Despite the potential, the evidence remains fragmented [17]. Studies vary in the type of technology, the type and dose of physical activity, and the quality of engagement metrics [16]. They also vary in outcomes and time horizons. There are interventions focused on mental health, others focused on consumption, and others with combined goals. There are also marked differences between interventions for young people and adults in a clinical context [19,20]. This heterogeneity makes it difficult to read in an integrated way and limits translation for programs and services [21].

Added to this are equity, privacy, and security challenges [22]. Digital literacy and access to devices are not uniform. In mental health and addictions, the collection and use of data can involve sensitive information, requiring transparency, data minimization, and clear governance. It is also necessary to consider unwanted effects, such as poorly timed messages, rigid goals that induce guilt, or gamification strategies that reduce intrinsic motivation [17,21].

In this framework, a comprehensive integrative review oriented towards implementation is needed. It is not enough to ask, “Is there effectiveness?”. It is important to understand “in whom”, “under what conditions” and “with what components”. It is also important to clarify how these interventions can be integrated into health promotion programs and clinical services, with quality and safety. Thus, this review aims to organize and critically synthesize the evidence on digital physical activity interventions for mental health promotion. In addition, we examine addiction-related outcomes as a clinically and public-health-relevant domain that frequently co-occurs with common mental health problems, integrating active components and implementation factors and providing operational recommendations for practice and research.

## 2. Methods

### 2.1. Design of This Review

A comprehensive integrative review was carried out, supported by structured research of the literature and explicit selection criteria. The aim was to critically synthesize the available evidence on digital interventions that promote physical activity with an impact on mental health outcomes and/or addictive behaviors, integrating results from studies with different methodological designs and implementation contexts.

### 2.2. Sources of Information

The search was conducted in biomedical, psychological, and multidisciplinary databases, including PubMed/MEDLINE, Scopus, Web of Science Core Collection, and PsycINFO. To capture interventions with a higher technology component, this research was complemented with technology-oriented databases, such as IEEE Xplore and/or ACM Digital Library, when relevant to wearables, sensors, and adaptive interventions.

### 2.3. Research Strategy

A search strategy based on four domains of terms was used:

1. Physical activity/exercise (e.g., physical activity, exercise, walking, aerobic, and resistance training);
2. Digital health and technologies (e.g., mHealth, mobile health, apps, smartphones, wearables, activity trackers, and digital interventions);
3. Mental health (e.g., depression, anxiety, stress, wellbeing, and sleep);
4. Addictive behaviors and relapse (e.g., addiction, substance use, craving, relapse, gaming, gambling, and problematic smartphone use).

The combinations of terms were adapted to each database. Boolean operators (AND/OR) and truncations were used when applicable. To maximize sensitivity, synonyms and close terms were included, maintaining specificity through the mandatory combination of (i) physical activity/exercise, (ii) digital components, and (iii) mental health outcomes and/or addictions.

The electronic search was complemented by a manual search of the reference lists of included studies (backward snowballing) and by identification of cited studies where appropriate (forward snowballing), with the aim of reducing the risk of missing relevant studies.

### 2.4. Time Period and Languages

Considering the evolution of the digital ecosystem and the maturation of mHealth, a main time window was defined from 2015 to 2026. English was used as the primary screening language due to its dominance in indexing and retrieval across the selected databases. No country-by-country restriction was applied.

### 2.5. Eligibility Criteria

#### 2.5.1. Inclusion Criteria

Studies that met all the following criteria were included:

- Intervention: Intervention with a digital component aimed at promoting physical activity and/or reducing sedentary lifestyle, including apps, platforms, wearables, SMS/notifications, digital tele-coaching, or adaptive interventions (e.g., JITAI/EMI).
- Population: Adolescents, young adults, or adults with (i) symptoms or diagnosis of mental health disorders (e.g., depression, anxiety, stress, or psychological distress) and/or (ii) addictive disorders/behaviors (substances and/or behavioral), or populations at relevant risk for relapse.
- Outcomes: Reporting at least one mental health outcome (e.g., depression, anxiety, stress, and well-being), and/or outcomes linked to addictions (e.g., craving, consumption, lapses/relapse, and retention in treatment), and/or complementary outcomes with clinical relevance (sleep, quality of life, and functioning).
- Type of publication: Empirical studies (qualitative or quantitative) and relevant reviews, when useful for contextualization and identification of gaps.

#### 2.5.2. Exclusion Criteria

The following were excluded:

- Studies in which the technology was used only for measurement/monitoring, without an interventional component.
- Digital interventions with no physical activity component (e.g., only digital psychotherapeutic content).
- Protocols without results were not included in the Results and were considered only in the Discussion to inform design trends and future directions, where particularly informative.
- Animal or laboratory studies with no relevance to health promotion in humans.

### 2.6. Study Selection Process

All records were exported to a bibliographic manager, and duplicates were removed. The selection took place in two phases:

1. Sorting by title and abstract, to exclude clearly irrelevant records;
2. Full-text evaluation, with systematic application of eligibility criteria.

Inclusion/exclusion decisions were recorded. Disagreements were resolved by consensus, with reassessment of the full text when necessary. The selection process was documented in such a way as to allow for the transparent presentation of the screening flow.

### 2.7. Data Extraction

An extraction grid with predefined dimensions was developed and applied consistently to the included studies:

- Identification (authors, year, and country) and context (clinical, community, and school/university);
- Study design (RCT, pragmatic, pre–post, pilot, implementation, or qualitative);
- Characterization of the sample (*n*, age, sex/gender when reported, clinical criteria, and comorbidities);
- Target condition (mental health, addictions, or combined) and setting (treatment/inpatient/outpatient/community);
- Core technology (app, wearable, tele-coaching, platform, or JITAI/EMI/EMA) and intervention architecture;
- Active components and behavioral change techniques (e.g., goals, self-monitoring, feedback, reinforcement, social support, gamification, coaching, or self-regulatory content);
- Prescription of physical activity (type, intensity when available, frequency, duration, progression, and supervision);
- Personalization (level, signals used, rules/decisions, micro-interventions, and timing);
- Outcomes and instruments (mental health, craving, consumption/relapse, sleep, quality of life, and functioning);
- Engagement and adherence (use, retention, participation in PA, abandonment, and acceptability);
- Implementation indicators (barriers/enablers, resources, fidelity, equity, and privacy/security).

For clarity, implementation fidelity was interpreted as the extent to which delivery adhered to intended content/dose and protocol reporting; progressive goals as stepwise increases in targets (e.g., steps/min/week) adapted over time; digital feedback as automated or human-mediated feedback derived from tracking data; and short follow-up as post-intervention only or ≤~3 months, when reported.

### 2.8. Data Summary

An integrative thematic synthesis was carried out, appropriate to the expected heterogeneity in the designs and metrics. Studies were organized by type of digital intervention (apps, wearables, tele-coaching/hybrids, or JITAI/EMI) and by primary objective (mental health promotion, risk reduction/relapse, or combined interventions).

The interpretation of the results privileged the following:

- Consistency of effects by outcome domain (mental health, craving/consumption, sleep, and quality of life);
- Relationship between active components and engagement/adherence;
- Implementation conditions associated with greater feasibility and sustainability (e.g., integration into services, intensity of human support, and technological accessibility).

When comparability between studies was limited, the results were presented in a narrative manner, with emphasis on the direction and plausibility of the effects, avoiding undue generalizations.

### 2.9. Considerations Regarding Methodological Quality

Although integrative reviews do not require formal risk-of-bias assessment, we did not conduct a full tool-based appraisal across all designs; instead, study design, clarity of intervention reporting, completeness of outcome reporting and follow-up were considered qualitatively to contextualize interpretation. These considerations were used only to contextualize interpretation in the Discussion and Limitations, not to exclude studies.

### 2.10. Ethical Considerations

Ethical approval was not required, since this review used only the published literature. However, relevant ethical implications for the topic were considered, namely, privacy, data governance and security in digital interventions aimed at vulnerable populations.

## 3. Results

The literature search identified studies evaluating digital interventions in which physical activity (or sedentary reduction) was a core component and that reported mental health and/or addiction-related outcomes. After duplicate removal and eligibility screening, the results synthesize evidence primarily from 16 empirical contributions (Refs. [23,24,25,26,27,28,29,30,31,32,33,34,35,36,37,38]), comprising 14 quantitative evaluations, one qualitative study, and one formative development paper, complemented by review-level contextualization only where explicitly indicated (cf. Ref. [39]). Consistent with the integrative scope, the study designs were heterogeneous (RCTs, feasibility/pilots, pre–post, and implementation-oriented evaluations), settings varied (clinical, workplace, community, and school/university), and follow-up reporting was inconsistent across studies.

In general, the studies were distributed in different contexts (clinical, community, and school/university) and by different age groups. There was a predominance of interventions focused on mobile apps [23,24,25], often complemented by wearables or digital self-monitoring strategies [26,27,28,40,41]. The follow-up time horizon tended to be short, with a lower proportion of studies reporting maintenance of effects after the end of the program [23,24,25,26,27,28,29,40].

### 3.1. Populations and Contexts of Intervention

Included populations ranged from individuals with symptoms of depression, anxiety, or stress [24,25,30,31,32] to populations with addictive disorders/behaviors, including substance use and, in some cases, behavioral dependencies [26,27,28,29,33]. In a clinical context, interventions were often integrated into broader programs, with remote or hybrid professional follow-up [26,27,28,29,34]. In a community and school/university context, the approach tended to privilege self-monitoring strategies, progressive goals and digital feedback [31,35,36].

Psychological comorbidity emerged as a cross-cutting aspect. In several studies, mental health outcomes were assessed in parallel with behavioral indicators (e.g., craving, drinking episodes, lapses, or behavioral proxies), which reinforces the rationale for integrated interventions [27].

### 3.2. Types of Digital Intervention

In the set analyzed, four dominant typologies emerged:

(i) Goal-focused and self-monitoring mobile apps.

These interventions included goal setting (e.g., steps/day or minutes/week), activity logging, and automatic feedback. In several cases, they incorporated psychoeducational content on stress, sleep and self-regulation [23,25,26,30,34].

(ii) Interventions with wearables and sensor-based feedback.

Wearables were used to track movement and, in some cases, sleep-related metrics. Feedback tended to be more frequent and “situational”, supporting goal adjustments and reinforcement of adherence. In some programs, the wearable component worked as an “objective test” and motivational element [27,29,37,40].

(iii) Hybrid models with human support.

In some clinical settings, interventions included human support to strengthen adherence, clarify objectives, and reduce barriers, complementing digital tools and/or self-monitoring. This support could take mild forms (check-ins, guidance, and follow-up during treatment), especially in populations with greater vulnerability and risk of abandonment [26,28,34].

(iv) Real-time adaptive interventions (JITAI/EMI).

These interventions seek to adapt content and timing based on cues (brief self-report, context, activity patterns, and sometimes sleep). In the corpus analyzed, real-time adaptation still appeared in a limited and heterogeneous way. The synthesis literature on JITAI in mobile interventions for physical activity reinforces the need to make explicit tailoring variables, decision rules, and implementation fidelity [16,18,21]. This guidance reflects framework-level synthesis and reporting recommendations, rather than consistent implementation across the included empirical digital physical activity interventions.

Across the included empirical studies, app- or web-based delivery was most frequent (*n* = 8; e.g., [23,24,25,26,30,31,36,38]). Wearable/pedometer-supported approaches were also common (*n* = 5; e.g., [27,29,34,37] and digital self-monitoring in addiction treatment settings [28]). Human support integrated with digital/self-monitoring (“hybrid” models) was present in a smaller subset (*n* = 2; e.g., peer-facilitated physical activity in opioid agonist therapy [28]; structured inpatient-linked pedometer intervention [34]). Explicitly, fully specified adaptive decision rules consistent with JITAI/EMI frameworks were rarely operationalized within digital physical activity interventions, and when discussed, were primarily informed by the framework-level literature rather than consistently implemented in the included empirical interventions [16,18,21].

### 3.3. Active Components and Behavioral Change Techniques

Most interventions used a common core of techniques: goal setting, self-monitoring, feedback, and reinforcement (cf. [39]). Planning components (action planning and coping), motivational messages and reminders were frequent. Social support was operationalized in different ways, from digital groups to progress-sharing features, although most studies did not use designs that allow incremental (component-level) benefit to be estimated [38].

Gamification appeared in some interventions, but with a variable design. When well-aligned with gradual goals and meaningful feedback, it can support engagement. When excessively competitive or rigidly performance-oriented, it can introduce friction in populations with greater symptomatology or emotional vulnerability and should be applied with caution.

### 3.4. Physical Activity Prescription: Type, Dose and Progression

The prescription tended to favor light-to-moderate aerobic activity (e.g., walking), often operationalized by steps/day [27,28,29,41]. There was a lower proportion of interventions with explicit prescription of intensity based on physiological parameters. Progression was generally incremental and adapted to previous performance, although it was not always described in detail.

When there was a supervised or tele-coaching component, there was greater detail in the prescription and greater attention to individual barriers, including fatigue, mood symptoms, and contextual constraints [36]. In populations with addictive behaviors, prescription was sometimes articulated with craving management and with alternative routines at critical times of the day [27].

### 3.5. Outcomes and Metrics: Mental Health, Addictions, Sleep and Engagement

Mental health outcomes included measures of depression, anxiety, stress, and psychological well-being [23,24,25,30,31,34,36,38]. In addictions, indicators of craving, consumption (frequency/quantity), lapses and, in some cases, relapse appeared [26,27,29]. Sleep appeared as a relevant outcome, but with variation in the form of measurement (self-report versus wearable metrics). Across studies, mental health outcomes were predominantly assessed via validated self-report scales, whereas activity was measured via device-based metrics (e.g., steps/minutes) and, in a smaller subset, passive sensing or model-based monitoring approaches.

Follow-up reporting was limited (often post-intervention only or ≤~3 months). Most studies reported immediate post-intervention outcomes, while fewer assessed maintenance beyond the program end [23,24,25,26,27,28,29,37,38,40]. Engagement metrics were heterogeneous and often not comparable across studies: several papers reported app-use indicators (e.g., logins and module completion), whereas others reported behavioral adherence (e.g., steps/minutes and participation), and only a minority reported both dimensions in a way that supports cross-study synthesis [26,30,34,38].

Cost reporting was sparse because cost variables were rarely reported. Across the included empirical studies, comprehensive data on equipment and personnel costs were rarely reported, limiting the possibility of summarizing cost ranges. As a pragmatic proxy for implementation burden, intervention architecture suggests lower resource requirements for app-only models, higher requirements when wearables are required, and the highest resource intensity when structured human support is integrated.

### 3.6. Personalization and JITAI: Signals, Decisions and Micro-Interventions

Personalization took place mainly at basic and intermediate levels. At a basic level, it adjusted goals based on past performance. At an intermediate level, it adapted content and recommendations based on reported preferences, barriers, and progress. In the corpus analyzed, advanced real-time personalization was less frequent and was not always described in sufficient detail to allow replication, reinforcing the importance of transparent reporting of adaptation logic and active components [23,24,25,30,36,39].

Recommendations tended to favor short and feasible actions (e.g., short walks, sedentary breaks, and simple exercises), with the adequacy of the context and the user’s ability being decisive for acceptance and adherence [39].

### 3.7. Implementation, Equity, Privacy and Security

In addition to outcomes, the literature highlighted implementation and equity challenges relevant to vulnerable populations. Implementation was conditioned by individual factors (literacy, motivation, and symptoms), technological factors (usability, friction, and reliability of sensors) and contextual factors (time, environment, and social support). Equity was critical, especially when access to smartphones, mobile data, or wearables was not guaranteed [42,43].

Data privacy and governance were particularly sensitive in this area. Collecting activity, sleep, and context patterns can be useful for personalization, but it requires transparency, minimization, and clear criteria for storage, access, and use [42,44]. In mental health and addictions, a “privacy-by-design” approach should be considered a requirement rather than optional.

### 3.8. Linking Intervention Components to Physical Activity Engagement/Adherence and Health Outcomes

Across the included empirical studies, attribution of effects to single components is constrained by the predominance of multicomponent designs and the limited use of dismantling or factorial approaches. Nevertheless, a descriptive mapping was possible where studies reported both (i) physical activity engagement/adherence (e.g., steps/minutes and participation) and (ii) mental health- and/or addiction-related outcomes. In these studies, goal setting combined with self-monitoring and actionable feedback constituted the most recurrent “backbone”, and was typically implemented with progressive targets and reminders, supporting engagement where reported (e.g., app/web-based programs in adults and college samples) [23,24,25,31,36]. Wearable/pedometer-supported approaches provided objective monitoring that enabled frequent feedback and goal adjustment; where step-based adherence was explicitly tracked, these designs supported engagement reporting and were linked to mental health outcomes in inpatient and youth contexts [34,37]. Across this subset, the most consistent pattern was that interventions reporting both engagement and outcomes typically combined (i) progressive goals, (ii) self-monitoring with objective or semi-objective metrics, and (iii) feedback that reduced decision burden. Conversely, because most studies were multicomponent and did not isolate ingredients, these patterns should be interpreted as co-occurring implementation features rather than evidence of the superiority of any single component.

In clinically vulnerable populations, low-friction execution and the presence of light human support (professional or peer-based) appeared to function as an enabling condition for participation and continuity, particularly in addiction treatment settings and inpatient-linked programs, where barriers and dropout risk are higher [26,28,34]. For addiction-related outcomes, evidence remained concentrated in alcohol treatment and methadone maintenance settings; these studies support feasibility and a plausible role for physical activity as a coping strategy, but controlled evidence with longer follow-up remains limited, constraining generalization to relapse prevention [26,27,28,29]. Overall, the current evidence supports implementation-oriented hypotheses that prioritize (i) progressive and feasible goals, (ii) objective or semi-objective self-monitoring, (iii) feedback that reduces decision burden, and (iv) scalable, low-intensity human support when targeting high-vulnerability contexts.

Importantly, only a subset of studies reported engagement/adherence alongside health outcomes in a way that supports cross-study comparison, and these links should, therefore, be interpreted as descriptive patterns rather than definitive component ‘effectiveness’. Where studies did not report engagement/adherence alongside outcomes, we did not infer component ‘success’ and treated such links as currently untestable within the available reporting.

## 4. Discussion

This comprehensive integrative review synthesizes evidence on digital interventions that promote physical activity with potential impact on mental health outcomes and/or addictive behaviors and organizes the field by technological typologies and intervention components. Three overarching messages emerge. Accordingly, the implementation recommendations presented should be interpreted as implementation-oriented hypotheses consistent with current evidence, rather than definitive effectiveness claims across all populations and settings.

First, most digital physical activity interventions rely on established behavioral change mechanisms (goal setting, self-monitoring, feedback, reinforcement, and planning) implemented through apps, web-based platforms, and sometimes wearables. This is consistent with broader evidence that physical activity can prevent or reduce anxiety and depressive symptoms and improve well-being, and with the recognized role of self-regulation processes in sustaining physical activity and reducing sedentary behavior [1,3,9,39]. Within digital formats specifically, narrative and meta-analytic evidence indicates that multicomponent “lifestyle” interventions can improve depression, anxiety, stress and well-being, although effect sizes and durability vary [45]. The implication is not that “technology causes improvement” per se, but that digital delivery can operationalize core behavioral change ingredients at scale, while supporting adherence through monitoring and feedback loops [12,13,14,39,45].

Second, when the focus shifts to addictive behaviors, the empirical base of interventions in which physical activity is the central therapeutic component and is digitally supported remains narrower and more context-specific than the mental health literature. Studies targeting alcohol use disorders illustrate a plausible pathway: wearable-supported lifestyle physical activity can be developed and delivered in treatment settings, and digital physical activity apps can be feasible for patients in alcohol treatment [26,27,29,33]. Evidence from methadone maintenance also suggests the feasibility of structured physical activity programs supported by digital self-monitoring (e.g., via trackers), including in populations with substantial vulnerability and complex clinical profiles [28]. However, in contrast with the mental health space, where multiple RCTs and workplace/community trials exist, the number of controlled trials in addictions with physical activity as the main intervention ingredient is still limited, and follow-up is often short [26,28,29,45]. This asymmetry should be acknowledged explicitly because it constrains how strongly we can generalize about relapse prevention effects driven by digital physical activity alone.

Third, advanced personalization, particularly architectures grounded in EMA/EMI and JITAI principles, appears more developed in the broader ecosystem of digital addiction interventions than in programs where physical activity is the central component. Conceptually, JITAIs are designed to deliver “the right support at the right time in the right context,” and frameworks emphasize decision rules, tailoring variables, and ongoing adaptation [18]. Systematic and qualitative syntheses show that in mental health, JITAI implementation is still evolving, with major gaps in operationalization, reporting, and real-world deployment [17,21]. In addictions, clinical digital recovery support services delivered via smartphone demonstrate the feasibility of self-managed, time-sensitive support, and systematic reviews summarize how EMI approaches can be used to reduce addictive behaviors [46,47]. In parallel, reviews and checklists of EMA/EMI studies highlight the importance of transparent reporting of tailoring logic and delivery characteristics; precisely because “personalization” can otherwise become a vague claim [44]. Together, these sources support a central opportunity: to translate the sophistication of EMA/EMI and JITAI design (well-articulated for addictions and mental health) into digital physical activity interventions, while keeping the behavioral prescription simple and safe [16,17,18,44,46,47].

### 4.1. Plausible Mechanisms and Why Digital Physical Activity May Matter

Physical activity effects on mental health and addiction-relevant processes are plausible and consistent with the outcome patterns reported in the reviewed studies. These pathways are presented as plausibility mechanisms consistent with the outcomes reported across the included studies, rather than as mechanistic findings directly tested within the reviewed interventions. At least four pathways are salient.

Stress regulation is central. The depression/anxiety relapse prevention literature indicates that stress-related pathways and maintenance processes are clinically meaningful, and physical activity may reduce stress reactivity while improving coping capacity [6,8]. In addictions, craving is a core mechanism linked to environmental cues and affective states, and an intervention that reduces negative affect or increases perceived control can plausibly reduce vulnerability to high-risk moments [7]. Sleep is another relevant pathway: physical activity has been associated with changes in sleep architecture and mood in naturalistic settings, supporting the rationale for integrating sleep-related targets or monitoring into behavior change interventions [8]. Affective pathways are also important: acute changes in tension and mood following activity can make physical activity a credible “microintervention” candidate, particularly in addictions where momentary risk fluctuates and is context-dependent [7,16,17,18]. Finally, self-efficacy and self-regulation support maintenance. Classic work on adherence highlights that sustaining physical activity is difficult, especially under distress and low support [10,11]. Cognitive control models further indicate that self-regulation processes influence physical activity and sedentary behavior, which aligns with using goal setting and feedback to build manageable routines [9,39].

These pathways interact. Stress and sleep can influence impulsivity and affective states, which, in turn, shape craving dynamics [7,8]. Therefore, interventions are more likely to be effective when they align their outcomes and delivery logic with a plausible mechanism (e.g., stress/craving coping vs. general lifestyle improvement) rather than treating “steps/day” as an end in itself [7,39].

### 4.2. What Seems to Work and Under What Conditions

Building on the descriptive mapping, implementation-oriented hypotheses are grounded in the subset of studies that reported both engagement/adherence indicators and health outcomes, while acknowledging that component-level causality cannot be inferred from multicomponent designs. Across the subset of studies reporting both engagement/adherence indicators and outcomes, three recurring implementation features were observed: (i) clear and gradual goals, (ii) self-monitoring with actionable feedback, and (iii) low friction in execution.

Goal setting is a consistent component across successful interventions. In digital physical activity programs for depression and related outcomes, RCT evidence shows that structured programs delivered via smartphone or web platforms can improve depressive symptoms, stress, psychological well-being, and quality of life when goals are feasible, and intervention content supports engagement [23,24,25,31,36]. In workplace and community-relevant contexts, application-based exercise has also been tested in clinical trials, with outcomes including depressive symptoms and burnout [38]. However, the field also shows heterogeneity in adherence and follow-up, reinforcing that the way targets are framed and adapted matters for sustainability [10,11,45].

Self-monitoring is beneficial when data are translated into decisions. Reviews of smartphone-based physical activity interventions for mental health emphasize that the “untapped potential” often lies in better tailoring of feedback and in aligning intervention prompts with user context and burden [39]. Emerging approaches also incorporate passive monitoring and predictive models for depression/anxiety signals in real-world users, suggesting a pathway for more context-aware delivery, although evidence is still early and feasibility-driven [30]. In practice, the distinction between “tracking” and “intervention” should be explicit: tracking alone is not sufficient; the intervention value emerges when monitoring supports self-regulation and timely action [12,39,44].

Low friction is decisive. Adherence limitations are well documented, and digital technologies are often framed as mechanisms to reduce access barriers [10,11,12,13]. In clinically vulnerable populations, structured support can stabilize routines. This appears particularly relevant in addictions, where feasibility trials of digital physical activity interventions in alcohol treatment and peer-facilitated approaches in methadone maintenance show that human support (professional or peer-based) can be part of the intervention architecture [26,27,28,29]. This aligns with the broader evidence that maintenance and relapse prevention require sustained processes rather than one-off gains [6,10,11].

### 4.3. Advanced Personalization: Opportunity, Risk, and Where Protocols Fit

Personalization should be treated pragmatically. There is a gradient between basic tailoring (adjusting goals based on past behavior), intermediate tailoring (adapting content to barriers/preferences), and advanced tailoring (EMA/EMI/JITAI). Frameworks emphasize that JITAIs require explicit specification of tailoring variables, decision points, and intervention options [18]. Yet, syntheses of JITAI in physical activity and mental health highlight persistent gaps in reporting, implementation fidelity, and translation to routine care [16,17,21]. In addictions, EMI approaches are increasingly systematized, with evidence from clinical trials of smartphone-based recovery support and systematic reviews of EMIs aimed at addictive behaviors [46,47]. Complementary work in SUD mHealth also points to rapid growth and to the need for careful consideration of engagement and clinical integration [43].

At the same time, advanced personalization introduces risks. On the one hand, complexity can reduce scalability, and on the other hand, poorly timed notifications may increase burden or distress [41]. In vulnerable populations, privacy and data governance become central, particularly when contextual and behavioral patterns are collected. Equity concerns also matter; access to devices, data plans, and wearables is not uniform, and JITAI implementation must be aligned with public health equity goals [22,43]. Here, protocols without results are best treated as “pipeline evidence”; they inform design trends (e.g., remote delivery and data-driven personalization) but should not be used as efficacy evidence in results. This distinction is consistent with reporting checklists and with the current maturity of the literature [44]. In practice, protocols and early-stage trials in depression/anxiety digital interventions can be used to frame future directions and methodological needs, not to support outcome claims [40,41,48,49].

Artificial intelligence (AI) may increasingly enable the operationalization of JITAI logic in digital physical activity interventions, particularly by improving real-time tailoring and reducing user burden. In practice, AI can support context-aware prompting (e.g., adapting timing and content based on recent activity patterns, sleep-related signals, or self-reported stress/craving) and risk-state inference to trigger feasible micro-actions when vulnerability is elevated. At the same time, AI-enabled tailoring raises implementation and governance challenges; decision rules and model behavior must be transparent enough to support clinical and public health accountability; bias and differential performance across subgroups must be assessed to avoid widening inequities; and data minimization, privacy-by-design, and secure handling of sensitive contextual signals are essential, especially in vulnerable populations. Consequently, staged evaluation is warranted (from robust “basic/intermediate” tailoring toward more adaptive AI-supported approaches) paired with explicit reporting of tailoring variables, decision logic, safety constraints, and equity/privacy safeguards.

### 4.4. Implications for Health Promotion and Services

Digital physical activity interventions may support short-term mental health promotion and potentially contribute to relapse prevention in selected settings if they are designed for adherence, safety, and implementation constraints. For clinical services, light hybrid models are plausible, including simple prescriptions, self-monitoring, and brief check-ins focused on barriers, coping, and routine building. This is aligned with feasibility evidence in addiction treatment contexts and with the broader adherence literature [10,26,28]. In addiction services, framing physical activity as a functional coping tool for stress and craving may be more acceptable than a performance-oriented framing [7,27]. For community and occupational contexts, scalability and equity should guide design choices; interventions dependent on expensive devices may exacerbate inequities, while smartphone-based designs can support broader reach [12,13,14,22,38].

For research, the priorities are clear: longer follow-up to test maintenance; minimal harmonization of outcomes (mental health, craving/consumption, sleep, quality of life, and engagement); and pragmatic or hybrid effectiveness–implementation designs [6,43,44,45]. In parallel, the field should progress into staged personalization, building robust basic/intermediate tailoring first and then testing more adaptive systems with explicit governance and risk assessment [18,22,44].

### 4.5. Limitations

The heterogeneity of interventions, technologies, outcomes and engagement measures limits direct comparisons and prevents strong generalizations. Digital adherence is often reported via app-use metrics, which may not reflect behavioral adherence to physical activity [39,44]. Follow-up is frequently short, limiting inference on maintenance and relapse prevention [10,45]. Moreover, evidence in addictions with physical activity as the central and digitally supported component is narrower than in mental health, requiring caution in interpretation and clear separation between efficacy claims and design recommendations [26,28,29,43]. Finally, equity, privacy and governance remain critical constraints, particularly when interventions rely on contextual data, passive sensing, or adaptive delivery [22,42,43,44].

Additionally, because we excluded studies where technology was used only for measurement/monitoring, we may have missed contexts in which behavior change occurs via mere measurement or observation effects (e.g., Hawthorne/observer effects), which should be considered when interpreting intervention-specific effects.

## 5. Conclusions

Digital interventions to promote physical activity are a promising avenue to support the promotion of mental health and, in selected contexts, contribute to the prevention of relapse into addictive behaviors. The available evidence suggests increased well-being mainly in depression, anxiety, stress and well-being, usually through multicomponent programs (goals, self-monitoring, and feedback). However, the heterogeneity of interventions and metrics, as well as the often-short follow-up, limit conclusions about maintenance.

In addiction-related outcomes, the evidence is more restricted and concentrated in some clinical settings but supports the rationale of physical activity as a functional coping strategy for stress, negative affect and craving. A future priority is to integrate EMA/EMI/JITAI principles into digital physical activity interventions, with pragmatic design, harmonized outcomes, and evaluation of implementation, privacy, and equity.

## Data Availability

No new data were created or analyzed in this study.

## Abbreviations

The following abbreviations are used in this manuscript:

AUD	Alcohol Use Disorder
EMA	Ecological Momentary Assessment
EMI	Ecological Momentary Intervention
JITAI	Just-In-Time Adaptive Intervention
mHealth	Mobile Health
RCT	Randomized Controlled Trial
SUD	Substance Use Disorder
